# Supplementary figures and images for: Modelling the impact of behavioural interventions during pandemics: A systematic review
Source: PLoS One. 2025 Feb 10;20(2):e0310611. doi: 10.1371/journal.pone.0310611 (PMC11809814; doi:10.1371/journal.pone.0310611)

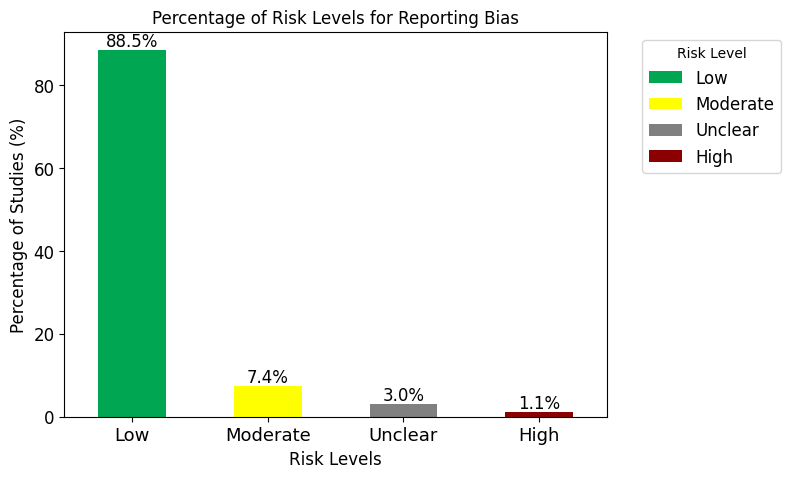

Supplement: S1 Fig — This figure shows the distribution of risk levels for reporting bias across the included studies. The risk levels are categorized as ‘Low,’ ‘Moderate,’ ‘Unclear,’ and ‘High,’ with corresponding percentages of 88.5%, 7.4%, 3.0%, and 1.1%, respectively. The risk levels are represented by green, yellow, gray, and red bars in the bar chart. (TIF) [file pone.0310611.s001.tif]

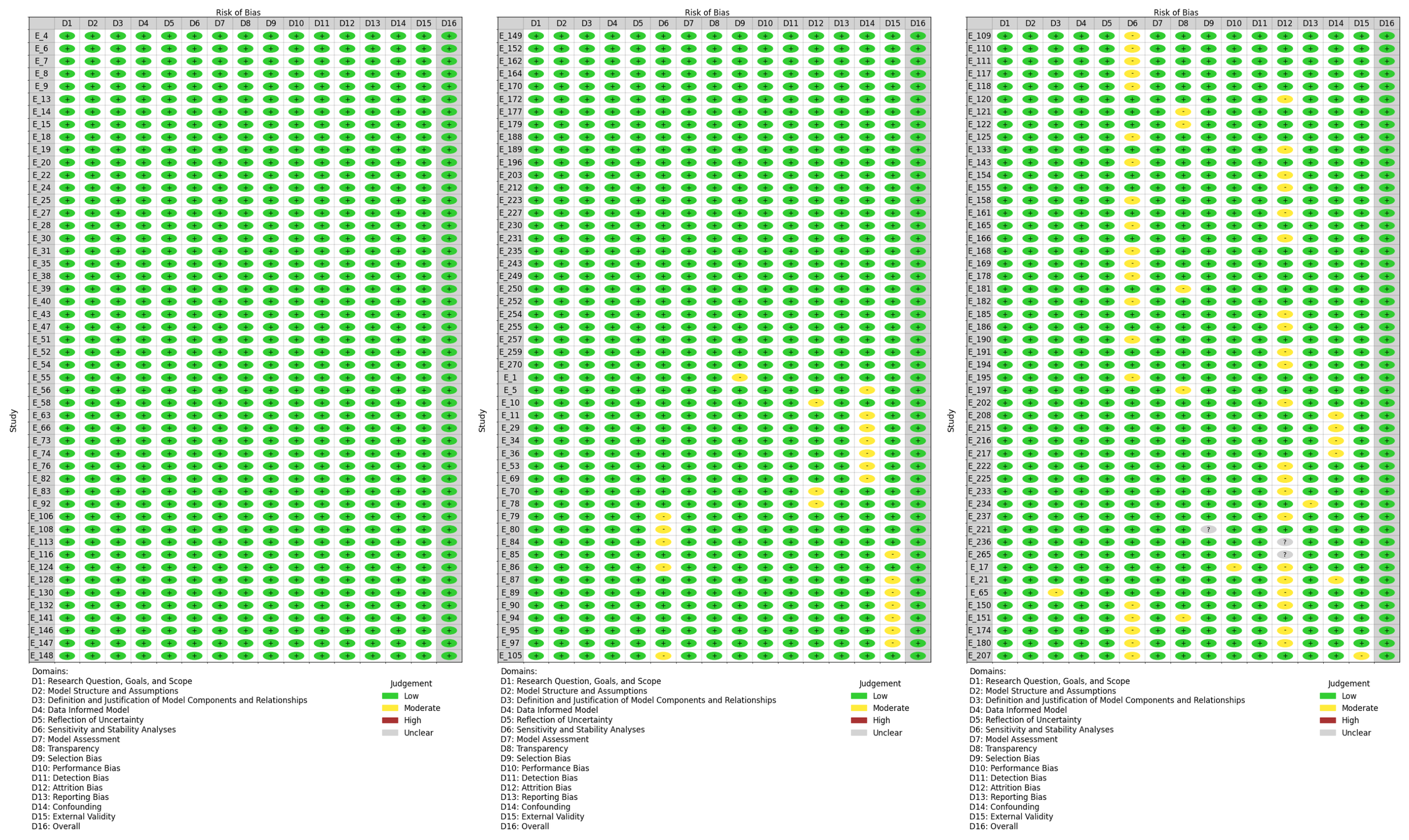

Supplement: S2 Fig — This figure presents a visual summary of the risk of bias assessment for studies included in Chunks 1, 2, and 3. The left panel (Chunk 1) represents the risk of bias assessment where all 50 articles have a low risk of bias. The assessment covers 15 domains, including ‘Research Question, Goals, and Scope,’ ‘Model Structure and Assumptions,’ ‘Data Informed Model,’ ‘Sensitivity and Stability Analyses,’ and others. Judgments are categorized as ‘Low,’ ‘Moderate,’ ‘High,’ or ‘Unclear’ risk of bias, and are represented by green, yellow, red, and gray symbols, respectively. (TIF) [file pone.0310611.s002.tif]

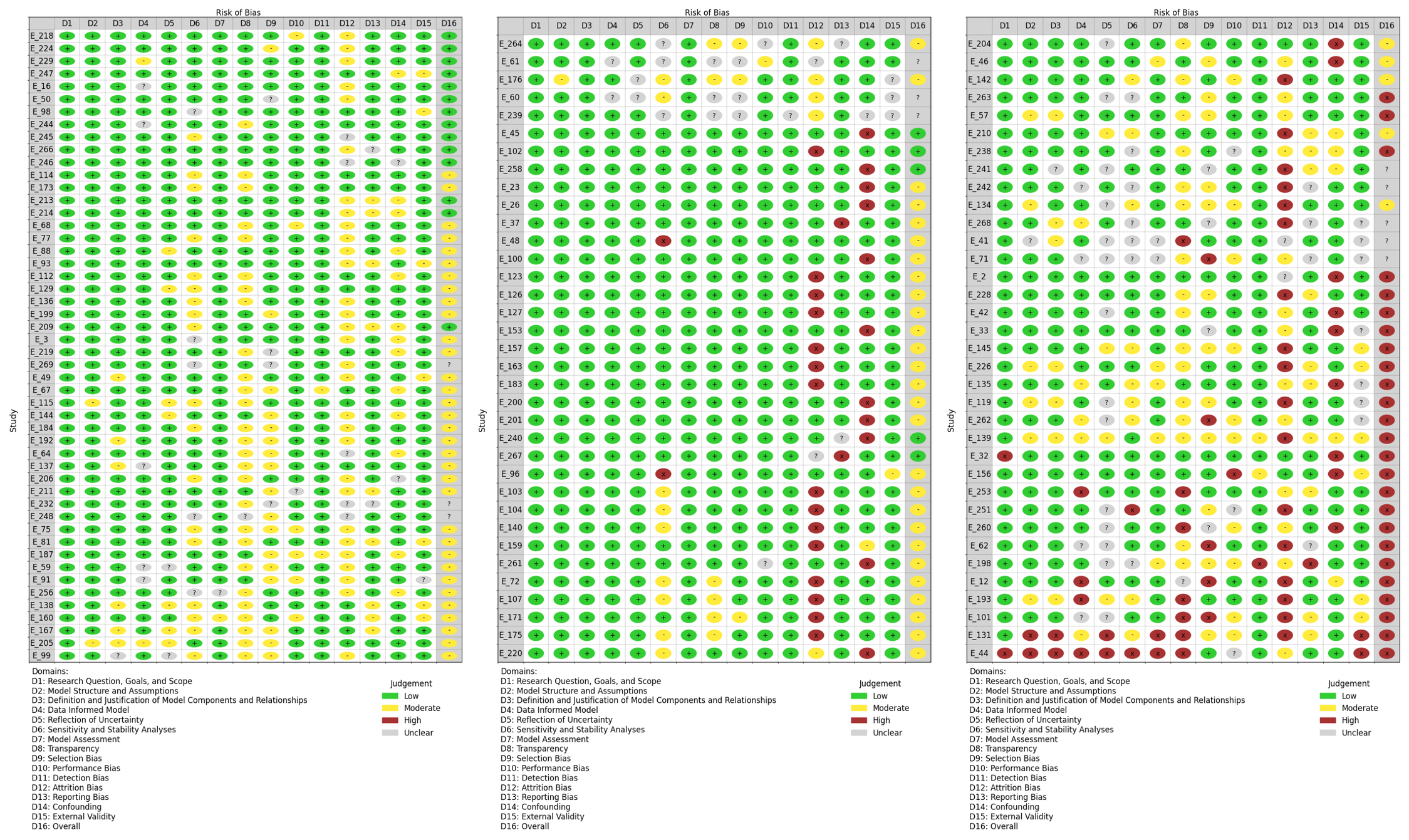

Supplement: S3 Fig — This figure presents a visual summary of the risk of bias assessment for studies included in Chunks 4, 5, and 6. The assessment is based on various domains, including ‘Research Question, Goals, and Scope,’ ‘Model Structure and Assumptions,’ ‘Data Informed Model,’ ‘Sensitivity and Stability Analyses,’ and others. Judgments are categorized as ‘Low,’ ‘Moderate,’ ‘High,’ or ‘Unclear’ risk of bias, and are represented by green, yellow, red, and gray symbols, respectively. (TIF) [file pone.0310611.s003.tif]

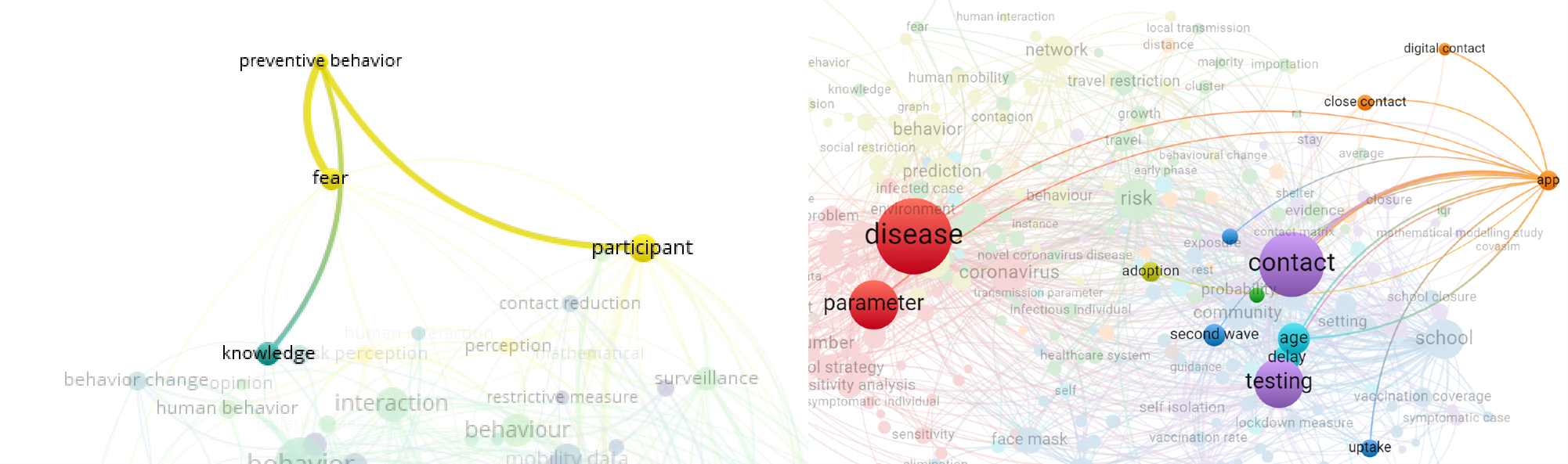

Supplement: S4 Fig — This figure presents a network visualization showing the relationships between the terms “preventive behavior” and “app.” The term “preventive behavior” (left) is linked with “fear,” “knowledge,” and “participant.” The term “app” (right) is linked with terms including “close contact,” “testing,” “digital contact,” and “contact”. (TIF) [file pone.0310611.s004.tif]

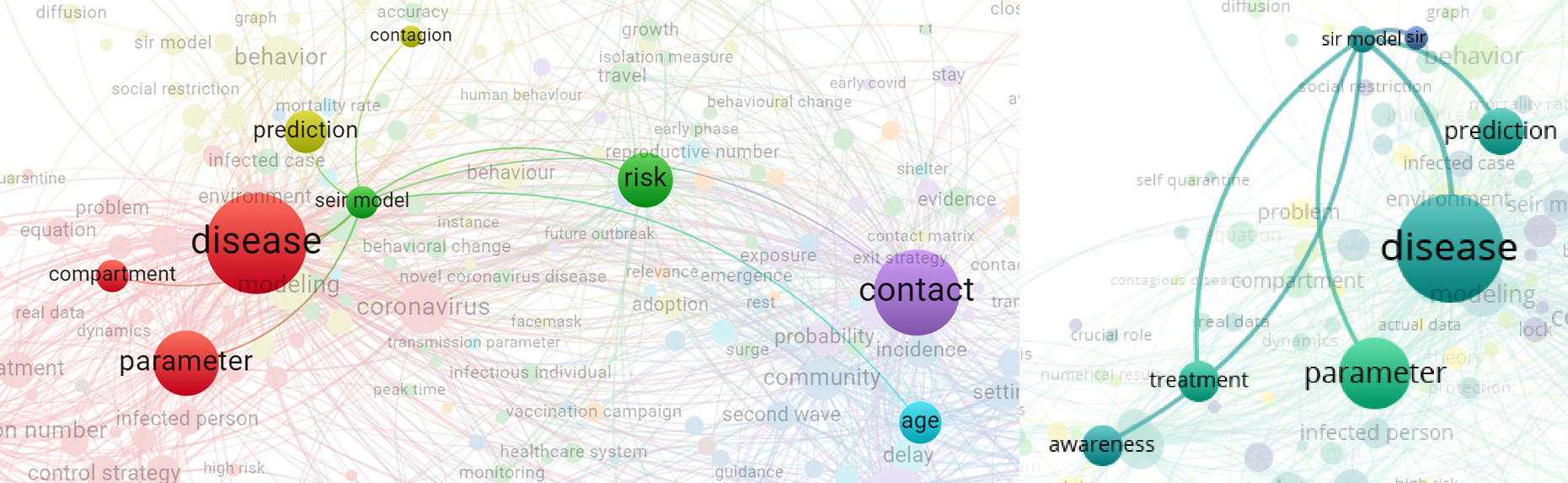

Supplement: S5 Fig — This figure displays network visualizations illustrating the relationships between terms associated with the “SEIR model” and “SIR model.” In the left panel, the “SEIR model” is linked with “parameter,” “disease,” “compartment,” “prediction,” and “contagion.” In the right panel, the “SIR model” is connected to similar terms, emphasizing its relationship with “disease,” “parameter,” “prediction,” and “treatment”. (TIF) [file pone.0310611.s005.tif]
