# Supplementary material for: Modelling the impact of behavioural interventions during pandemics: A systematic review
Source: PLoS One. 2025 Feb 10;20(2):e0310611. doi: 10.1371/journal.pone.0310611 (PMC11809814; doi:10.1371/journal.pone.0310611)
Supplement: S1 Appendix — The search strategy consisting search keywords. (PDF) [file pone.0310611.s006.pdf]

S1 Appendix. Search strategy. The search strategy focused on the following keywords:

> '("COVID*" OR "corona*")' AND '("Model*")' AND '("Transmission Model*" OR "Compartmental Model*" OR "Population Dynamic*" OR "Epidemiological Model*" OR "Mathematical Model*")'

This search query was used across the four databases: PsycInfo, MEDLINE, Psychology and Behavioural Sciences Collection, and MathSciNet.

In the case of EMBASE, the search query was slightly modified. The single quotation marks ('()') that encompassed the brackets in the original query were removed. Instead of the double quotation marks used in the original query to enclose each term (e.g., "behavio*"), a single quotation was employed. The adjusted query for EMBASE is as follows:

> ('COVID*' OR 'corona*') AND ('Model*') AND ('Transmission Model*' OR 'Compartmental Model*' OR 'Population Dynamic*' OR 'Epidemiological Model*' OR "Mathematical Model*')

This modification ensures compatibility with EMBASE's search syntax while retaining the essence of the original query.

For Web of Science, a modified search string/query was used. It is adjusted to the search syntax of this database. It is as follows:

> (Ti=("COVID*" OR "corona*") OR AB=("COVID*" OR "corona*") OR KP=("COVID*" OR "corona*")) AND (TI=("Model*") OR AB=("Model*") OR KP=("Model*")) AND (TI=("Transmission Model*" OR "Compartmental Model*" OR "Population Dynamic*" OR "Epidemiological Model*" OR "Mathematical Model*") OR AB=("Transmission Model*" OR "Compartmental Model*" OR "Population Dynamic*" OR "Epidemiological Model*" OR "Mathematical Model*") OR KP=("Transmission Model*" OR "Compartmental Model*" OR "Population Dynamic*" OR "Epidemiological Model*" OR "Mathematical Model*"))

The search query for Scopus followed a modified version based on the database's search syntax. It is as follows:

> (TITLE-ABS-KEY("COVID*" OR "corona*") AND TITLE-ABS-KEY("Model*") AND TITLE-ABS-KEY("Transmission Model*" OR "Compartmental Model*" OR "Population Dynamic*" OR "Epidemiological Model*" OR "Mathematical Model*"))
