# Supplementary material for: Modelling the impact of behavioural interventions during pandemics: A systematic review
Source: PLoS One. 2025 Feb 10;20(2):e0310611. doi: 10.1371/journal.pone.0310611 (PMC11809814; doi:10.1371/journal.pone.0310611)
Supplement: S1 Table — This table represents extraction items, their descriptions, and the possible values. (PDF) [file pone.0310611.s009.pdf]

S1 Table. Extraction Items, Descriptions, and Possible Values

No	Extraction Item	Description	Possible Values
1	Authors	Names of the authors of the study.	Author name/s as string
2	Study ID(doi)	Unique identifier for each study	Numeric or alphanumeric string
3	Title	Title of the research paper.	Title as a string
4	Publication year	Year of publication of the study	Year
5	Study location	Country level	Country names (e.g., USA, Brazil, India)
6	Model Name	Specify the model name used	SEIR model, Compartmental model, logistic regression, etc.
7	Study design	Specify the study design (e.g., a randomized controlled trial (RCT), observational study, modeling study, etc.)	Experimental, Quasi-experimental, Observational
8	Sample size	Size of the study population	Numeric value or range (e.g., $500, > 1000$)
9	Type of data	Type of data collected (Primary, secondary, experimental, etc.)	Primary data, secondary data, experimental data, or other
10	Target population	Population group targeted by the behavioural intervention	General population, Healthcare workers, High-risk groups
11	Setting	Where the study took place.	Setting details (e.g., community, Urban, Rural, healthcare setting)
12	Intervention type	Specific behavioural intervention implemented	Social distancing, Hand hygiene, Mask wearing
13	Outcome measure	Metric used to assess the impact of the intervention	Number of COVID-19 cases, Number of COVID-19 deaths, Proportion of the population with protective behaviors
14	Basic Reproduction Number(R0)	Estimated value of R0 (if reported).	e.g., $3.4, 4, 1.18$
15	Effective Reproduction Number(Reff or Rc)	Average value of the reported effective reproduction number, Reff or Rc, (if reported, 'NA' if not).	e.g., $0.8, 0.5, 0.68$ or NA
16	Outcome measure results	Observed effect of the intervention on the outcome measure	Significant decrease, Non-significant decrease, No change, Significant increase
17	Key Findings	Summarize the main findings related to the impact of behavioural interventions on pandemic outcomes.	Positive impact on reducing COVID-19 transmission, Mixed results, Limited evidence of effectiveness, Inconclusive findings
18	Additional Comments	Additional notes or comments relevant to the study.	Methodological challenges noted, Data collection issues, Not applicable
19	Exact population size consideration(N=0 as a small setting, N=1 as the whole population)	Fill with "Yes=1" if the article uses the population of the specified study area or "No=0"* if the study used small units representing the sample size/population size (e.g., the population of doctors = 150, nurses = 50, technical staffs = 20)	$1, 0$
20	Compartmental Model?(Yes=1, No=0)	Fill with "Yes=1" if the study used a compartmental model and "No=0" if not	$1, 0$
21	Open Access(Yes=1, No=0)	Fill with "Yes=1" if the study is open access and "No=0" if not	$1, 0$
22	Quality Assessment	Evaluation of the study's methodological quality.	High, Medium, Low, Unclear
