# Supplementary material for: Modelling the impact of behavioural interventions during pandemics: A systematic review
Source: PLoS One. 2025 Feb 10;20(2):e0310611. doi: 10.1371/journal.pone.0310611 (PMC11809814; doi:10.1371/journal.pone.0310611)
Supplement: S2 Table — This table represents the description of the 15 risks of bias assessment domains where eight are from the Principles for good practice in modeling and simulation and the rest seven are from the ROBINS-I. (PDF) [file pone.0310611.s010.pdf]

S2 Table. Principles for good practice in modeling and simulation and Domains from the ROBINS-I (Risk Of Bias In Non-randomized Studies - of Interventions) tool (The Netherlands, 2024)

**No.**	**Principles for good practice**	**Description**
1	Research Question, Goals, and Scope	The research question, modeling goals, and the model's scope should be clearly defined.
2	Model Structure and Assumptions	The model structure and assumptions should be explained and justified.
3	Definition and Justification of Model Components and Relationships	Model components and their relationships should be defined. The chosen relationships between model components should be justified.
4	Data Informed Model	The model should be informed by data. Data selection, analysis, and interpretation should be aligned with the research question and the model's scope; data sources should be described.
5	Reflection of Uncertainty	The model should reflect uncertainty in inputs.
6	Sensitivity and Stability Analyses	Sensitivity analyses (to assess the influence of model inputs) and stability analyses (to evaluate the impact of modeling decisions) should be undertaken and reported.
7	Model Assessment	Models should be assessed for their ability to address the research question within the stated scope.
8	Transparency	Modeling methods should be transparent. Adequate details about the structure, data, and assessment methods should be reported so that the modeling process is replicable.
Domains from the ROBINS-I (Risk Of Bias In Non-randomized Studies - of Interventions) tool		
**No.**	**Domains**	**Description**
1	Selection Bias	Evaluating how data sources were selected and whether they were representative of the population and context.
2	Performance Bias	Identifying any factors that could have influenced the model's performance in a biased manner.
3	Detection Bias	Assessing how outcomes were measured and detected, ensuring consistent and objective criteria.
4	Attrition Bias	Reviewing any excluded or lost data points and their potential impact on results.
5	Reporting Bias	Checking for selective reporting of results and ensuring all relevant outcomes were reported.
6	Confounding	Identifying and controlling for potential confounding factors.
7	External Validity	Assessing the generalizability of the findings to other populations, settings, and times.
	Overall	
