# Supplementary material for: Modelling the impact of behavioural interventions during pandemics: A systematic review
Source: PLoS One. 2025 Feb 10;20(2):e0310611. doi: 10.1371/journal.pone.0310611 (PMC11809814; doi:10.1371/journal.pone.0310611)
Supplement: S3 Table — (PDF) [file pone.0310611.s011.pdf]

S3 Table. Descriptive statistics of categorical variables before the risk of bias assessment

No	Variable	Category	Frequency	%
1	Study design	Modeling and simulation study	384	94.6%
		Predictive modeling study	15	3.7%
		Observational study	7	1.7%
2	Type of data	Secondary data	381	93.8%
		Primary data	16	3.9%
		Experimental data	5	1.2%
		Mixed data	4	1%
3	Year	2020	165	40.640
		2021	130	32.020
		2022	111	27.340
4	Continent	Africa	16	3.941
		Asia	148	36.453
		Europe	110	27.094
		Australia (Oceania)	4	0.985
		North America	81	19.951
		South America	31	7.635
		Worldwide	16	3.941
5	Population consideration	No	67	16.502
		Yes	339	83.498
6	Compartmental model	No	43	10.591
		Yes	363	89.409
7	Open Access	No	154	37.931
		Yes	252	62.069
		**Total**	406	100%
