# Supplementary material for: Modelling the impact of behavioural interventions during pandemics: A systematic review
Source: PLoS One. 2025 Feb 10;20(2):e0310611. doi: 10.1371/journal.pone.0310611 (PMC11809814; doi:10.1371/journal.pone.0310611)
Supplement: S4 Table — (PDF) [file pone.0310611.s012.pdf]

S4 Table. Summary statistics of the regression model parameters and 95% confidence intervals including sample size variable. Residual std. error: 1.734 (df = 236, 69 observations deleted due to missingness), Multiple R^2 : 0.07431, Adjusted R^2 : 0.01548, F-statistic: 1.263 ($df = 15, 236$, p-value: 0.2267).

**No.**	**Variable**	**Category**	**Estimate**	**Std. Error**	**t value**	**Pr(> |t|)**	95% CI	
							**2.5%**	**97.5%**
1	(Intercept)		2.959	1.934	1.530	0.1273	−0.851	6.769
2	Population Consideration	No (Ref.)						
		Yes	−0.445	0.353	−1.262	0.2082	−1.139	0.250
3	Compartmental	No (Ref.)						
		Yes	0.235	0.657	0.358	0.7207	−1.059	1.530
4	Type data	Experimental data (Ref.)						
		Mixed data	−2.149	2.136	−1.006	0.3154	−6.357	2.059
		Primary data	−0.943	2.036	−0.463	0.6439	−4.954	3.069
		Secondary data	−0.997	1.785	−0.559	0.5770	−4.513	2.519
5	Study design	Modeling and simulation study (Ref.)						
		Observational study	−2.218	1.399	−1.585	0.1143	−4.974	0.538
		Predictive modeling study	−1.085	1.204	−0.901	0.3684	−3.456	1.287
6	Continent	Africa (Ref.)						
		Asia	0.806	0.518	1.554	0.1215	−0.216	1.827
		Australia (Oceania)	0.342	1.806	0.189	0.8501	−3.216	3.899
		Europe	0.921	0.538	1.713	0.0880	−0.138	1.980
		North America	1.188	0.546	2.177	0.0305 *	0.113	2.264
		South America	1.287	0.618	2.081	0.0385 *	0.069	2.505
		Worldwide	1.343	0.779	1.725	0.0859	−0.191	2.878
7	Open access	No (Ref.)						
		Yes	0.436	0.242	1.800	0.0732	−0.041	0.912
8	Sample size (*ni*)		−0.000	0.000	−0.576	0.5654	−0.000	0.000

*Significant at 5% level. – Ref. indicates for a reference category.
