# Supplementary material for: Modelling the impact of behavioural interventions during pandemics: A systematic review
Source: PLoS One. 2025 Feb 10;20(2):e0310611. doi: 10.1371/journal.pone.0310611 (PMC11809814; doi:10.1371/journal.pone.0310611)
Supplement: S5 Table — (PDF) [file pone.0310611.s013.pdf]

S5 Table. Variance Inflation Factors (VIF) for predictors in the model to check for multicollinearity

**No.**	**Variable**	**GVIF**	**Df**	GVIF^(1/(2*Df))
1	Population Consideration	1.274596	1	1.128980
2	Compartmental	1.898605	1	1.377899
3	Type of data	2.777630	3	1.185621
4	Study design	2.754469	2	1.288278
5	Continent	1.357670	6	1.025808
6	Open access	1.142368	1	1.068816
7	Sample size (ni)	1.010923	1	1.005447
