# Supplementary material for: Modelling the impact of behavioural interventions during pandemics: A systematic review
Source: PLoS One. 2025 Feb 10;20(2):e0310611. doi: 10.1371/journal.pone.0310611 (PMC11809814; doi:10.1371/journal.pone.0310611)
Supplement: S6 Table — (PDF) [file pone.0310611.s014.pdf]

S6 Table. Frequency table for each domain of the risk of bias (ROB) assessment

No.	Domain	Low	Moderate	High	Unclear
		$N(\%)$	$N(\%)$	$N(\%)$	$N(\%)$
1	Research Question, Goals, and Scope	268 (99.26%)	0 (0%)	2 (0.74%)	0 (0%)
2	Model Structure and Assumptions	258 (95.56%)	9 (3.33%)	2 (0.74%)	1 (0.37%)
3	Definition and Justification of Model Components and Relationships	252 (93.33%)	14 (5.19%)	2 (0.74%)	2 (0.74%)
4	Data Informed Model	248 (91.85%)	7 (2.59%)	4 (1.48%)	11 (4.07%)
5	Reflection of Uncertainty	239 (88.52%)	11 (4.07%)	2 (0.74%)	18 (6.67%)
6	Sensitivity and Stability Analyses	190 (70.37%)	61 (22.59%)	4 (1.48%)	15 (5.56%)
7	Model Assessment	259 (95.93%)	6 (2.22%)	2 (0.74%)	3 (1.11%)
8	Transparency	204 (75.56%)	54 (20.00%)	7 (2.59%)	5 (1.85%)
9	Selection Bias	220 (81.48%)	33 (12.22%)	5 (1.85%)	12 (4.44%)
10	Performance Bias	245 (90.74%)	18 (6.67%)	1 (0.37%)	6 (2.22%)
11	Detection Bias	264 (97.78%)	4 (1.48%)	1 (0.37%)	1 (0.37%)
12	Attrition Bias	155 (57.41%)	72 (26.67%)	32 (11.85%)	11 (4.07%)
13	Reporting Bias	239 (88.52%)	20 (7.41%)	3 (1.11%)	8 (2.96%)
14	Confounding	216 (80.00%)	31 (11.48%)	20 (7.41%)	3 (1.11%)
15	External Validity	235 (87.04%)	22 (8.15%)	2 (0.74%)	11 (4.07%)
16	Overall	169 (62.59%)	65 (24.07%)	25 (9.26%)	11 (4.07%)
