# Supplementary material for: Modelling the impact of behavioural interventions during pandemics: A systematic review
Source: PLoS One. 2025 Feb 10;20(2):e0310611. doi: 10.1371/journal.pone.0310611 (PMC11809814; doi:10.1371/journal.pone.0310611)
Supplement: S7 Table — (PDF) [file pone.0310611.s015.pdf]

S7 Table. An overall rating criteria of the risk of bias assessments.

No.	Overall Risk of Bias	Interpretation	Within a Study
1	Low risk of bias	Plausible bias unlikely to seriously alter the results.	Low risk of bias for all key (important) domains.
2	Moderate risk of bias	Plausible bias that moderately weakens confidence in the results.	Moderate for at least two important domains or only one high but all low domains.
3	High risk of bias	Plausible bias that seriously weakens confidence in the results.	High risk of bias for at least two key domains or combined high risks in several domains or moderate risks in 8 or more domains.
4	Unclear risk of bias	Plausible bias that raises some doubt about the results.	Unclear risk of bias for two or more key (important) domains or combined unclear risks in several domains.
