# Supplementary material for: Modelling the impact of behavioural interventions during pandemics: A systematic review
Source: PLoS One. 2025 Feb 10;20(2):e0310611. doi: 10.1371/journal.pone.0310611 (PMC11809814; doi:10.1371/journal.pone.0310611)
Supplement: Attachment — Submitted filename: Response to Reviewers_CJA_TKG.pdf [file pone.0310611.s020.pdf]

Martial L Ndeffo-Mbah, Ph.D

Academic Editor

PLOS ONE

Dear Dr. Ndeffo-Mbah and reviewers,

Thank you for providing detailed feedback on our manuscript titled "Modelling the impact of behavioural interventions during pandemics: A systematic review" (Manuscript ID: PONE-D-24-38089). We appreciate the time and effort you and the reviewers have taken to assess our work. In response to the comments, we have made several changes to the manuscript as requested.

Below is a detailed point-by-point response to the comments raised by the reviewers and academic editor. For clarity, the reviewers' comments are provided in bold, followed by our responses and the corresponding changes made in the manuscript. Please note that all line numbers refer to the original manuscript, as the revisions and tracked changes have changed the line numbering in the updated version. Also note that due to the use of `latexdiff` for generating the marked-up (tracked changes) version, some tables may appear partially hidden or misaligned. The clean (unmarked) version of the manuscript accurately reflects the intended formatting and content of all tables.

Response to Editor's Comments

Comment 1: Journal requirements:
When submitting your revision, we need you to address these additional requirements.

1. Please ensure that your manuscript meets PLOS ONE's style requirements, including those for file naming. The PLOS ONE style templates can be found at `PLOSOne_formatting_sample_main_body.pdf` and

   `PLOSOne_formatting_sample_title_authors_affiliations.pdf`

   Response: Thank you for noting the importance of adhering to PLOS ONE's style requirements. We have carefully reviewed and formatted our manuscript according to the PLOS ONE style templates provided.

2. When completing the data availability statement of the submission form, you indicated that you will make your data available on acceptance. We strongly recommend all authors decide on a data sharing plan before acceptance, as the process can be lengthy and hold up publication timelines. Please note that, though access restrictions are acceptable now, your entire data will need to be made freely accessible if your manuscript is accepted for publication. This policy applies to all data except where public deposition would breach compliance with the protocol approved by your research ethics board. If you are unable to adhere to our open data policy, please kindly revise your statement to explain your reasoning and we will seek the editor's input on an exemption. Please be assured that, once you have provided your new statement, the assessment of your exemption will not hold up the peer review process.

   Response: Thank you for emphasizing the importance of a clear data-sharing plan. We confirm that all data are fully available without restriction on the Open Science Framework (OSF) at the following view-only link: https://osf.io/dy9ck/?view˙onlyData. The included files as listed in the response for **Comment 3.1**.

3. As required by our policy on Data Availability, please ensure your manuscript or supplementary information includes the following:

   Comment 3.1:

   – A numbered table of all studies identified in the literature search, including those that were excluded from the analyses.
   – For every excluded study, the table should list the reason(s) for exclusion.
   – If any of the included studies are unpublished, include a link (URL) to the primary source or detailed information about how the content can be accessed.
   – A table of all data extracted from the primary research sources for the systematic review and/or meta-analysis. The table must include the following information for each study:
   – Name of data extractors and date of data extraction
   – Confirmation that the study was eligible to be included in the review.
   – All data extracted from each study for the reported systematic review and/or meta-analysis that would be needed to replicate your analyses.
   – If data or supporting information were obtained from another source (e.g. correspondence with the author of the original research article), please provide the source of data and dates on which the data/information were obtained by your research group.
   – If applicable for your analysis, a table showing the completed risk of bias and quality/certainty assessments for each study or outcome. Please ensure this is

provided for each domain or parameter assessed. For example, if you used the Cochrane risk-of-bias tool for randomized trials, provide answers to each of the signalling questions for each study. If you used GRADE to assess certainty of evidence, provide judgements about each of the quality of evidence factor. This should be provided for each outcome.

Response:: All data are available on the Open Science Framework (OSF) at the following view-only link: https://osf.io/dy9ck/?view˙onlyData. All data are in Excel file format. The following datasets are included:

1) A numbered table of all studies identified in the literature search is available as "TKG-CJAallSearch_15781.xlsx".
2) The $7,616$ articles screened are available including those that were excluded from the analyses with a column that stores reason for exclusion or inclusion as "All_7616_Articles_w_Reason.xlsx".
3) A table of all data extracted from the primary research sources for the systematic review and/or meta-analysis is available under the name "TKG-CJA-ExtractedData_406_270.xlsx".
4) A table showing the completed risk of bias and quality/certainty assessments for each study or outcome is provided as ''270ROBexcel_Nov11cor.xlsx".

Comment 3.2: An explanation of how missing data were handled.
This information can be included in the main text, supplementary information, or relevant data repository. Please note that providing these underlying data is a requirement for publication in this journal, and if these data are not provided your manuscript might be rejected.

Response: Thank you for the reminder. An explanation of how missing data were handled is included in the manuscript.

Additional Editor Comments:
The manuscript require a in-depth revision from both a methodological and a results standpoint. Systematic review generally requires a critical appraisal of the included studies, but this seems to be lacking. Morover, the analysis seems to be mostly focused around the reproductive number (basic and effective), so the scope of the study may need to be refined to enphasize its focus and limitation to these metrics.

Response: Thank you for your feedback. We appreciate the importance of clarity and depth in both methodology and results. We carefully followed the PRISMA 2020 guidelines and conducted a comprehensive bias assessment using combined criteria from ROBINS-I and modeling principles, ensuring that each study was evaluated across multiple domains, including transparency, attrition, and confounding factors. Our extensive risk-of-bias assessment across 15 domains demonstrates that many studies scored low to moderate in risk, reflecting the methodological rigor of this review. As per your suggestion, we have also made adjustments to the manuscript to strengthen these methodological details. In terms of results, we prioritized the basic reproduction number (R_0) as a primary outcome due to its significance in transmission dynamics and since we considered it for quantitative synthesis. However, we also considered other key metrics such as COVID-19 cases, transmission rates, and deaths in the narrative analysis. We believe the manuscript meets the required methodological and results presentation depth.

General Change Made: Based on the feedback received, we have focused solely on R_0 in the results section of our revised manuscript, excluding summary statistics of R_c (or R_{eff}) from Tables 3 and 4 as an outcome measure, given that R_c fluctuates over time and typically fluctuates around one, which limits its utility as a static outcome measure.

Additionally, any interpretation of R_c (or R_{eff}) as a single, static outcome measure has been removed from the manuscript.

Response to Reviewers

Reviewer #1 Comments

Comment: The authors conducted a systematic review in accordance with PRISMA 2020 guidelines to explore transmission models and assess the impact of behavioral interventions on COVID-19 outcomes. They included multiple peer-reviewed studies focusing on human subjects and modeling behavioral interventions during the pandemic, utilizing real data from various geographical regions. The methodologies employed in the studies are clearly articulated, and the presentation of the findings is commendable. Results are presented in detail and enhanced with visual representations. This thorough review underscores the significance of behavioral interventions in mitigating COVID-19 transmission and identifies opportunities for enhancing the transparency and rigor of future research.

This work can be accepted for publication. However, there is one point on which a brief discussion will be appreciated:

While a thorough descriptive and narrative analysis is described in the study, can you provide some rationale on the variation in the reproduction number across countries/ continents? From this systematic review, what is that major takeaway in this context? Why could there be this variability? Will data quality and analysis have an impact on this?

Response: Thank you for these valuable suggestions.

1. **Variation in reproduction numbers across countries:** We agree with the reviewer's observation that the variation in reproduction numbers across different regions is a key point. As detailed in the "Results of Individual Studies" subsection and supported by Tables 3 and 4 along with the accompanying narrative analysis, the manuscript addresses the variability of R_0 due to regional and intervention-related factors. Table 3 provides a descriptive summary across studies, illustrating the range of R_0 values. In contrast, Table 4 demonstrates geographic differences by continent, highlighting how variations in location and interventions contribute to observed differences in transmission rates.
2. **Conclusion of major takeaways:** The major takeaway from this systematic review is that the effectiveness of behavioral interventions in reducing transmission is highly context-dependent, underscoring the importance of tailoring interventions to specific settings rather than relying on a one-size-fits-all approach. We already indicated this in the "Discussion and Conclusion" section under "Common results," where we highlight how the effectiveness of interventions like distancing, quarantine, and lockdowns can vary significantly based on the timing, intensity, and specific settings in which they are implemented. Additionally, the Narrative Analysis section in the Results addresses this theme by showing that the effectiveness of interventions can differ based on factors such as age groups targeted, intervention timing, and combinations of strategies, reinforcing the context-dependent nature of these measures.

General Change Made: Based on the feedback received, we have focused solely on R_0 in the results section of our revised manuscript, excluding summary statistics of R_c (or R_{eff}) from Tables 3 and 4 as an outcome measure, given that R_c fluctuates over time and typically fluctuates around one, which limits its utility as a static outcome measure. Additionally, any interpretation of R_c (or R_{eff}) as a single, static outcome measure has been removed from the manuscript.

Reviewer #2 Comments

Comment 1: Overall, this paper is important for health behavioral science. I appreciated the effort that the authors put in to examine this research question that will undoubtably be important for future pandemics. The additional risk of bias assessment was interesting and I think adds another dimension to this manuscript that is not always addressed in the literature. I think this manuscript it written well and is important for outbreak investigation science and epidemiology.

Methods
General: The analyses and dedication to reporting as much as possible is commendable for such a large amount of data. I think the different analyses and acknowledgement and transparency of methodology is great.

Response: Thank you very much for your positive feedback on the significance of our work and the value of the risk of bias assessment. We are pleased to hear that you find our manuscript well-constructed and relevant to both outbreak investigation science and epidemiology.

Comment 2: Specific Line-by-Line Comments

- **Line 49**: Capitalize "Behavioral" at the beginning of this sentence to match the other bullet points.
- **Line 537**: The term 'eradicate' may not be appropriate here. I think 'eliminate' is a better choice.
- **Lines 537 and 543**: Starting a sentence with the reference number does not seem appropriate.
- **Line 574**: The 0 in the R0 term is not subscripted here.
- **Line 693**: Why is there an upside-down exclamation point here? Is this supposed to be '60%'?
- **Line 702**: Missing a quotation mark after the word lockdown.
- **Line 745**: Why are some of these terms using double quotations and some have single quotations? Was this on purpose, and if yes, what is the reason?

  Tables
  Table 4 is very very interesting. I think this table particularly will be very important for future analyses and pandemic research.

Response: Thank you for the detailed suggestions. We have adapted the manuscript based on these comments. We appreciate your positive feedback regarding Table 4. We are glad to hear it has relevance and potential impact for future research.

Comment 3: Figures
I don't know if this is a function of the PDF I downloaded to review the article—but the figures are not highest resolution and somewhat blurry. Please ensure that all of the

figures are the correct resolution when submitting.
Figure 11 is very difficult to read.....if there is a way to enlarge it or make it much higher quality, that is necessary.

I would also consider decreasing the number of figures—there are a couple that are also in Table form within the manuscript that repeat information. Although this is more of a personal choice, I think the number of figures can be reduced.

Response: Thank you for your helpful feedback on the figures.

1. **Resolution Improvement**: Initially, all figures were in .png format and then converted to .tiff for submission. To ensure optimal resolution, we have now generated all figures directly in .tif format from the original data, which should improve clarity in the final submission.
2. **Specific Notes on Figure 11**: Figure 11 is a composite visualization created by merging four network visualizations centered around key intervention terms: "face mask", "self-isolation", "media campaign", and "physical distancing". Each of these figures was generated in VOSviewer by using the mouse pointer to highlight each term individually, displaying closely related terms connected within the network. This approach allows the visualization to illustrate the interconnections and prominent relationships associated with each intervention across studies, making the context and influence of these specific terms clearer. Due to software limitations, further resolution enhancement may not be possible. However, we ensured that the original figure's resolution was maximized for submission quality.
3. **Reduction of Figures**: We have taken your feedback into account and excluded Figure 7, which had some overlap with the information presented in Tables 3 and 4. Hence, its removal should enhance clarity without sacrificing essential information.

General Change Made: Based on the feedback received, we have focused solely on R_0 in the results section of our revised manuscript, excluding summary statistics of R_c (or R_{eff}) from Tables 3 and 4 as an outcome measure, given that R_c fluctuates over time and typically fluctuates around one, which limits its utility as a static outcome measure. Additionally, any interpretation of R_c (or R_{eff}) as a single, static outcome measure has been removed from the manuscript.

Reviewer #3 Comments

Comment: This paper has an interesting premise, which would be a good addition to the literature. However, there are substantial issues that should be addressed before publication. These include major inconsistencies and contradictions throughout, results presented without methods, many methods that are insufficiently detailed for reproducibility, practices or methods that do not adhere to systematic review best practices, and a lack of clarity that appears to be due to unfamiliarity with infectious disease modeling. Furthermore, the primary stated objectives were not addressed.

Response: Thank you for your thoughtful review and your interest in our paper's contribution to the literature. We appreciate your constructive feedback and would like to address the concerns raised.

- **Clarification on Contradictions and Inconsistencies**: We acknowledge your comment regarding contradictions and inconsistencies throughout the manuscript. However, without specific examples, it is challenging to identify and address these

points effectively. Any detailed guidance you could provide on areas where contradictions may appear would be vital in refining our manuscript.

– **Reproducibility and Methodological Detail**: We have aimed to provide sufficient methodological detail to ensure reproducibility, in line with systematic review best practices. We appreciate the feedback on reproducibility and are open to any specific suggestions you might have that would further enhance the clarity or depth of our methods section.

– **Adherence to Systematic Review Best Practices**: We have conducted this review following the PRISMA 2020 guidelines and we intend to adhere strictly to systematic review best practices. If there are particular aspects of the methodology that appear to deviate from these standards, we would be grateful for specific points to address and improve upon.

– **Primary Objectives and Study Scope**: Our manuscript aims to address the primary objectives as outlined in the introduction, focusing on the effectiveness of behavioral interventions on COVID-19 outcomes. We carefully structured the analysis and results to align with these objectives. If there are specific points where the alignment between objectives and findings could be strengthened, we would appreciate your suggestions.

General Change Made: Based on the feedback received, we have focused solely on R_0 in the results section of our revised manuscript, excluding summary statistics of R_c (or R_{eff}) from Tables 3 and 4 as an outcome measure, given that R_c fluctuates over time and typically fluctuates around one, which limits its utility as a static outcome measure. Additionally, any interpretation of R_c (or R_{eff}) as a single, static outcome measure has been removed from the manuscript.

Reviewer #4 Comments

Comment 1: This systematic review aims to synthesize available transmission modeling literature on the impact of behavioral interventions on COVID-19 outcomes. The authors present variation in the 1) basic reproduction number and 2) effective reproductive number after intervention, both among all included studies and stratified by geographic region, and describe the impact of behavioral interventions on other COVID-19 outcomes. They also characterize the landscape of included publications, including the type of model used and type of behavioral intervention explored. Finally, they assess the risk of bias of included publications across multiple criteria.

Strengths of this analysis include the authors' comprehensive literature search, multifaceted approach to synthesizing results from heterogeneous studies, and risk of bias assessment. The methods are described in detail and analyses appear to have been performed to a high technical standard.

Response: Thank you for your positive feedback on the strengths of our systematic review. We appreciate your acknowledgment and will carefully address your specific comments to further improve the manuscript.

Comment 2: A weakness is the regression modeling analysis of predictors of R_0. The authors should revise the introduction section to be more focused and add citations for comparisons with existing literature in the discussion. In general, suggest revising the manuscript to be more concise.

Response: Thank you for your detailed comments. We have revised the introduction to streamline our focus and removed general points not directly related to our study's objectives. We also edited the regression modeling analysis.

Comments: In the introduction, suggest zooming in on the focus of the paper more quickly. For example,

> **Comment 3:** lines $6 - 8$ may be true, but are distracting as the paper is not about building a comprehensive social safety net.
>
> **Response:** We have removed the lines on social safety nets (lines 6-8) and modified the idea.
>
> **Comment 4:** Lines 12-17: Meaning of social and behavioral indicators is unclear. Also the paper does not discuss how to build models or conduct modeling: suggest revising to motivate study objectives.
>
> **Response:** Lines 12-17 have been revised to clarify our motivation.
>
> **Comment 5:** Regression analysis results (starting on line 500). Inclusion of other variables would likely make this analysis more informative.
>
> – Lines 307-309, say "We considered the following independent variables: population consideration, compartmental, type of data, sample size, study design, and study location (continent)." Why didn't the final model include variables such as sample size and type of population?
>
>   **Response:** Thank you for this valuable comment, which allowed us to provide a clearer, step-by-step rationale for our model selection. Initially, we included seven variables: population consideration, compartmental, type of data, sample size, study design, continent, and open access. The results from this full model, incorporating all seven predictors, can be found in `S4 Table` in the `Supporting Information`. In this initial model, the F-statistic was not significant, and the coefficient for the sample size variable was close to zero, indicating minimal predictive value. Consequently, we reran the model excluding the sample size variable while retaining the other six variables, and this revised model yielded a significant F-statistic. Furthermore, multicollinearity was assessed through Variance Inflation Factors (VIF), and no multicollinearity issues were identified (see `S5 Table` in the `Supporting Information`). Based on these findings, we chose to present the model excluding sample size in the main paper (`Table 5`), while details of the model including sample size are provided in the supporting information.
>
> – Table 5 and lines 508-516: Please include 95% confidence intervals for coefficients.
>
>   **Response:** We have now added the 95% confidence intervals for the coefficients in Table 5.
>
> **Comment 6:** Discussion section lacks citations and does not effectively integrate results with existing literature.
>
> – Lines 830-834: SEIR models are particularly effective compared to what? Regression models? SIR models? Network models? I agree that compartmental models are useful, but they are not necessarily the best choice in all situations.
>
>   **Response:** Thank you for your comment. Our intention was to emphasize that SEIR models are effective at capturing disease transmission dynamics and intervention effects due to their compartmental structure. However, we recognize that other models, such as agent-based, regression or network models, can also be valuable in specific contexts. We have revised the text to make it clear that SEIR models are a robust choice for capturing epidemic dynamics but do not imply superiority over alternative approaches in all scenarios.

– 850-851: Need citations for previous studies.

Response: We have included the citation for this part.

– 855: Need citations for other global studies.

Response: We have included the citation for this part.

– 859: Again, need citations for other studies.

Response: We have included the citation for this part.

– Lines 863-864: Need citations.

Response: We have included the citation for this part.

– Lines 867-873: This paragraph is repeating vs. interpreting the results.

Response: We have revised the paragraph to focus on interpreting the results rather than restating them.

– Lines 875-896: This paragraph repeatedly mentions ”some studies”. Suggest making this more quantitative.

Generally not concise, which distracts from the main message. Suggest editing for concision and clarity.

Response: We have edited lines 875-896.

MINOR ISSUES

– Line 15: Please define ”social and behavioral interventions” when first mentioned. You give examples of interventions, but not until line 49.

Response: Thank you for this suggestion. We have revised line 15.

– Also, suggest standardizing which interventions you are considering across lines 45-46, 50-51, and 70-71.

Response: Thank you for your suggestion. Our study encompasses a broad range of interventions, including but not limited to hand washing, mask-wearing, social distancing, and contact tracing.

– Lines 53-54: What does ”with practice, without practice” mean in this context?

Response: The phrase ”with practice, without practice” refers to situations where interventions, such as social distancing or mask-wearing, are either implemented consistently (”with practice”) or not implemented at all or inconsistently (”without practice”). To avoid ambiguity, we have revised the text to specify this.

– Lines 201-202: Suggest referencing this supporting information above where the eight principles and seven domains are first mentioned.

Response: Thank you for the suggestion. We have referenced the sources of the eight principles and seven domains on lines 194 and 198 in the manuscript. Additionally, we specify in line 201 that further details on these principles and domains are provided in the S2 Table within the Supporting Information.

– Lines 207-208: Unclear what you mean by ”we synthesized the effect measures based on the qualitative and quantitative data reported in the studies.” Suggest either specifying examples such as percent reduction here or removing this part of the sentence and describing it in later bullets.

Response: We have revised the text in lines 207-208 to clarify our approach. Specifically, we used narrative synthesis to report both qualitative and quantitative outcomes as they were stated in each study. For example, if a study reported a 20% reduction in death rate, this outcome was included in our extracted data as provided by the original authors.

– Table 1: Please clarify what you mean by ”modeling and simulation study” vs. ”predictive modeling study”.

Response: Thank you for your comment. We have clarified the distinction between "modeling and simulation study" and "predictive modeling study" in Table 1. In our classification:

✓ Modeling and Simulation Study refers to studies using models like compartmental models (e.g., SEIR) that simulate epidemic progression under various scenarios, often to examine disease dynamics.

✓ Predictive Modeling Study refers to studies that apply predictive techniques, such as logistic regression, aimed at forecasting specific outcomes rather than simulating processes over time.

– Figures 7-8: Please list continents in the same order for these two figures. Also, might be helpful to include sample size counts in Figure 8.

Response: Thank you for your feedback. Based on feedback from other reviewers, and because it closely resembles the information already presented in Tables 3 and 4, we have decided to exclude the original Figure 7. The original Figure 8, now renamed Figure 7, has been updated to include the sample size counts for each continent based on your suggestion.

– Lines 416-417: Do you have evidence/citations that the countries listed (US, China, India, Brazil, Italy) have large national science budgets? Might be true but not discussed. Also, this might be better in discussion.

Response: We agree that providing evidence for the national science budgets of these countries would strengthen the statement. We have now added a citation to support this assertion.

– Figure 6: How did you combine the individual bias domains into an overall bias score?

Response: Thank you for your question regarding the approach to combining individual bias domains into an overall bias score in Figure 6. We have clarified our methodology in the Methods section under the sub-subsection, "Rating the overall risk of bias level."

In line with Cochrane guidelines (Higgins et al., 2019), the overall risk of bias is determined by assessing the relative importance of each domain, with a focus on critical domains such as "Research Question, Goals, and Scope" (D1), "Data-Informed Model" (D4), "Attrition Bias" (D12), and "Confounding" (D14), which have a significant influence on study reliability. An overall high-risk judgment is assigned when two or more of these critical domains are rated high risk. High risks across multiple domains also indicate that the study results may lack reliability. We have provided additional details in the S7 Table of Supporting Information.

– Figure 11: How did you decide which terms to highlight in this figure?

Response: Thank you for your question. Figure 11 is a composite visualization created by merging four network visualizations, each centered around key intervention terms: "face mask," "self-isolation," "media campaign," and "physical distancing." Each network visualization was generated in VOSviewer by manually highlighting these terms with the mouse pointer, which displayed closely related terms connected within the network. This approach enabled us to illustrate the prominent relationships and interconnections associated with each intervention across studies, providing a clearer context for the influence of these specific terms.

– Line 768: I suspect that sample size may also be a main contributor to the variability.

Response: Thank you. We have revised the text to include sample size as an additional factor contributing to this variability.

OTHER POINTS

- Initials in the financial disclosure don't match any of the authors' names.

  Response: This work was funded by the ZonMW grant, and we did not receive any other funding. We made sure that in the revision, all initials match our names.

General Change Made: Based on the feedback received, we have focused solely on R_0 in the results section of our revised manuscript, excluding summary statistics of R_c (or R_{eff}) from Tables 3 and 4 as an outcome measure, given that R_c fluctuates over time and typically fluctuates around one, which limits its utility as a static outcome measure. Additionally, any interpretation of R_c (or R_{eff}) as a single, static outcome measure has been removed from the manuscript.

Once again, we would like to thank you for your detailed and constructive feedback. We hope that the revisions made have addressed all of your concerns, and we look forward to hearing your response.

Sincerely,
Tsega Kahsay Gebretekle
Also on behalf of co-author Casper Albers
