# Supplementary material for: Modelling the impact of behavioural interventions during pandemics: A systematic review
Source: PLoS One. 2025 Feb 10;20(2):e0310611. doi: 10.1371/journal.pone.0310611 (PMC11809814; doi:10.1371/journal.pone.0310611)
Supplement: Attachment — Submitted filename: Response to Reviewers_CJA_TKG2.pdf [file pone.0310611.s021.pdf]

Martial L Ndeffo-Mbah, Ph.D

Academic Editor

PLOS ONE

Dear Dr. Ndeffo-Mbah and reviewers,

Thank you again for providing detailed feedback on our manuscript titled "Modelling the impact of behavioural interventions during pandemics: A systematic review" (Manuscript ID: PONE-D-24-38089). We appreciate the time and effort you and the reviewers have taken to assess our work. In response to the comments, we have addressed the minor issues raised by reviewer #4 as requested.

The response is described in the next page. The comment is provided, followed by our responses and the corresponding changes made in the manuscript. Please note that all line numbers refer to the original manuscript, as the revisions and tracked changes have changed the line numbering in the updated version.

Response to Reviewers

Reviewer #4 Comments

Comment: Thank you for all your work to revise the manuscript and respond to suggestions. I appreciate your explanation for the regression model selection process. A few last minor recommendations for the "Regression analysis results for R_0" section:

- When mentioning significant F-statistics, please state the null hypothesis (which model are you comparing to).
- Note on presenting AIC/BIC in your tables: these are useful for comparing between models. A single value on its own is not interpretable.
- Suggest removing text about statistical significance of intercept (lines 565-566 in revised manuscript).

Response: Thank you for these valuable suggestions.

- We now state that the null hypothesis tests whether the regression model provides a significantly better fit compared to a baseline model that includes no predictors. This clarification has been added in the "Regression analysis results for R_0" section.
- We agree with the reviewer's point regarding presenting AIC/BIC in tables. Since these metrics are primarily useful for comparing between models, and we are not performing model comparisons, we have excluded the AIC/BIC from the table in the revised manuscript. We also excluded them from the "S4 Table" of the "Supporting Information" section.
- We agree with the reviewer's point and have removed the text about the statistical significance of intercept in the regression results section.

Once again, we would like to thank you for your detailed and constructive feedback. We hope that the revisions made have addressed all of your concerns, and we look forward to hearing your response.

Sincerely,
Tsega Kahsay Gebretekle
Also on behalf of co-author Casper Albers
